# Understanding Omicron: Transmissibility, immune evasion and antiviral intervention

**DOI:** 10.1002/ctm2.839

**Published:** 2022-05-11

**Authors:** Markus Hoffmann, Prerna Arora, Stefan Pöhlmann

**Affiliations:** ^1^ Infection Biology Unit, German Primate Center – Leibniz Institute for Primate Research Göttingen Germany; ^2^ Faculty of Biology and Psychology Georg‐August‐University Göttingen Göttingen Germany

**Keywords:** antibody, omicron, SARS‐CoV‐2, spike

1

The severe acute respiratory syndrome coronavirus 2 Omicron variant (B.1.1.529) swept the globe with breathtaking speed, rapidly displacing the Delta variant and causing record numbers of new infections. Many questions regarding the Omicron variant are open, including its origin, which may reside in animals, immunocompromised human patients, or secluded human populations, and potential differences in the biological properties of the Omicron sublineages, BA.1, BA.2, and BA.3. Here, we will comment on host cell interactions governing cell entry of the Omicron variant and their implications for transmissibility, virulence, immune evasion, and therapeutic options. Our focus will be on the viral spike (S) protein, which facilitates viral entry into cells and is the key target of the neutralizing antibody response. We will address the following questions:

## 2 IS THE OMICRON VARIANT LESS DANGEROUS AS COMPARED TO PREVIOUS VARIANTS?

On the one hand, the Omicron variant evades neutralizing antibodies with unprecedented efficiency.^1^ On the other hand, Omicron, frequently hits populations with a high percentage of convalescent or vaccinated individuals and, as compared to previous variants, is more adept at infecting such individuals. Thus, due to preexisting immunity many Omicron patients have a reduced propensity to develop severe disease. Further, the Omicron variant exhibits a reduced intrinsic virulence – that is its capacity to cause disease in immunological naïve patients is diminished.^2^ The net result of these opposite effects is a reduced severity of the Omicron variant and apparently, there are no apparent differences between BA.1 and BA.2. However, it should be noted that the reduction in intrinsic virulence is probably low, as underlined by the high number of severe cases within the elderly population in Hong Kong, which has a low vaccination rate.

## 3 WHAT IS THE REASON FOR THE REDUCED INTRINSIC VIRULENCE OF THE OMICRON VARIANT?

The viral spike (S) protein might hold answers. Compared to previously circulating variants the Omicron variant fuses cells (a function of the S protein) with reduced efficiency and it has been suggested that this might contribute to the reduced virulence. Further, in contrast to all previously circulating variants, Omicron prefers the cellular protease Cathepsin L over TMPRSS2 for S protein activation.^3^ Cathepsin L is required for the spread of the Omicron variant in nasal epithelium while a TMPRSS2‐related protease supports spread in the trachea^3^, ^4^ but neither seems to be available in the alveoli,^4^ which might limit replication and capacity of the Omicron variant to induce severe disease.

## 4 WHY IS THE OMICRON VARIANT HIGHLY TRANSMISSIBLE?

It has been speculated that usage of Cathepsin L, which is more widely available in the nasal epithelium than TMPRSS2, might promote the establishment of primary infection. However, the Omicron variant does not seem to replicate to higher levels in the nasal epithelium as compared to other severe acute respiratory syndrome coronavirus 2 (SARS‐CoV‐2) variants.^5^ The factors limiting replication remain to be explored and increased sensitivity to innate defences might be one scenario. In this regard, the impact of the restriction factors IFITM2/3, which specifically block Cathepsin L‐dependent entry will be of interest. Finally, it is noteworthy that BA.2 is becoming dominant in several countries due to a fitness advantage and not because of increased immune evasion. What confers that fitness advantage to BA.2 remains to be elucidated.

## 5 ARE PEOPLE WHO RECOVERED FROM INFECTION BY PREVIOUSLY CIRCULATING SARS‐COV‐2 VARIANTS PROTECTED AGAINST THE OMICRON VARIANT?

Sera from individuals infected with SARS‐CoV‐2 variants Alpha, Beta or Delta show variable but robust cross‐neutralization against each of the three variants. In contrast, neutralization of the Omicron variant is dramatically reduced,^1^ indicating that immunity following infection by previously circulating SARS‐CoV‐2 variants provides little to no protection against the Omicron variant. Indeed, an increased risk of reinfection in South Africa was associated with the Omicron but not the Beta or Delta variants.^6^

## 6 DO VACCINES PROTECT AGAINST THE OMICRON VARIANT?

Partial evasion of vaccination‐induced neutralizing antibodies has been reported for several SARS‐CoV‐2 variants before, however, not to the extent that the Omicron variant is able to.^1^ While two‐dose vaccination regimens with the messenger RNA (mRNA) or vectored vaccines induce higher neutralizing antibody titers compared to whole‐inactivated virus or single‐dose adenovirus‐based vaccines, neutralization of the Omicron variant is generally low. Heterologous two‐dose vaccination with adenovirus‐based (first dose) and mRNA‐based vaccines (second dose) as well as triple vaccination with mRNA vaccines have been shown to substantially increase neutralization of the Omicron variant.^1^ In keeping with these findings, two shots of mRNA vaccine provided reduced protection against symptomatic Omicron as compared to Delta infection while triple vaccination installed robust protection against severe disease upon infection with both variants.^7^ However, it remains to be determined how long protection lasts. Finally, Omicron specific‐derivatives of the mRNA vaccines are being developed but it is at present unknown whether they will do better than the originals. Results from studies examining booster immunization in animal models suggest that they might not.

## 7 WHICH ANTIVIRAL TREATMENT OPTIONS ARE SUITABLE FOR THERAPY OF PATIENTS INFECTED WITH THE OMICRON VARIANT?

Treatment with recombinant monoclonal antibodies (mAbs) that neutralize SARS‐CoV‐2 can reduce the risk for severe disease. Most of the antibodies approved for coronavirus disease 2019 (COVID‐19) therapy are poor neutralizers of the Omicron variant and the ones that neutralize efficiently do so in a subvariant‐specific fashion: Sotrovimab (S309) efficiently neutralizes BA.1 but not BA.2 while Cilgavimab shows the reverse phenotype.^1^, ^8^ In contrast, Bebtelovimab potently neutralizes both BA.1 and BA.2.^8^ Sotrovimab, Cilgavimab and Bebtelovimab thus constitute viable treatment options for Omicron patients but the success of Sotrovimab or Cilgavimab therapy will depend on the Omicron subvariant, suggesting that a cocktail of these antibodies should be used. Antiviral drugs that target the virus directly and are in clinical use (e.g. Remdesivir, Molnupiravir and Nirmatrelvir) are active against the Omicron variant^9^ and no subvariant‐specific differences were reported so far.

## 8 WHAT IS NEXT?

Subvariants of Omicron might evolve and dominate the pandemic as is currently the case for BA.2. These variants might recombine with other variants, as recently reported for Omicron and Delta variants. Alternatively, variants that presently circulate at a low level, as well as novel variants, may come to dominance in the future. Such a scenario is supported by the observation that infection of naïve individuals with the Omicron subvariant BA.1 induced humoral responses which neutralized BA.1 to some extent but exerted little neutralizing activity against other variants.^10^

## CONFLICT OF INTEREST

The authors declare no conflict of interest.
